# Chagas Disease Treatment and Rational Drug Discovery: A Challenge That Remains

**DOI:** 10.3389/fphar.2019.00873

**Published:** 2019-08-02

**Authors:** Ana Catarina Cristovão Silva, Maria Carolina Accioly Brelaz-de-Castro, Ana Cristina Lima Leite, Valéria Rêgo Alves Pereira, Marcelo Zaldini Hernandes

**Affiliations:** ^1^Laboratório de Imunopatologia e Biologia Molecular, Departamento de Imunologia, Instituto Aggeu Magalhães, Recife, Brazil; ^2^Laboratório de Parasitologia, Centro Acadêmico de Vitória, Universidade Federal de Pernambuco, Vitória de Santo Antão, Brazil; ^3^Departamento de Ciências Farmacêuticas, Universidade Federal de Pernambuco, Recife, Brazil; ^4^Programa de Pós-graduação em Inovação Terapêutica, Centro de Biociências, Universidade Federal de Pernambuco, Recife, Brazil

**Keywords:** Chagas disease, drug development, drug target, Trypanosoma cruzi, benzonidazole

Chagas disease is a neglected disease caused by *Trypanosoma cruzi*, a protozoan of the family Trypanosomatidae. Chagas disease is an important public health issue, affecting mainly populations who have lower socioeconomic resources and less access to health. Estimates suggest that 6 to 7 million people worldwide have the disease and that it is predominantly distributed among the 21 countries of Latin America, where more than 1 million of those cases occur in Brazil (WHO, 2015).

Since its discovery in 1909, several compounds have been tested as candidates for its treatment, such as arsenic, fuchsin, bismuth, antihistamines, amphotericin B, antibiotics, and others (Coura and De Castro, 2002). Currently, the treatment is based on the administration of two drugs: benzonidazole (**Figure 1A**) or nifurtimox (**Figure 1B**). These drugs are more effective in the acute phase over the chronic phase and have a high toxicity rate (Morilla and Romero, 2015).

**Figure 1:**
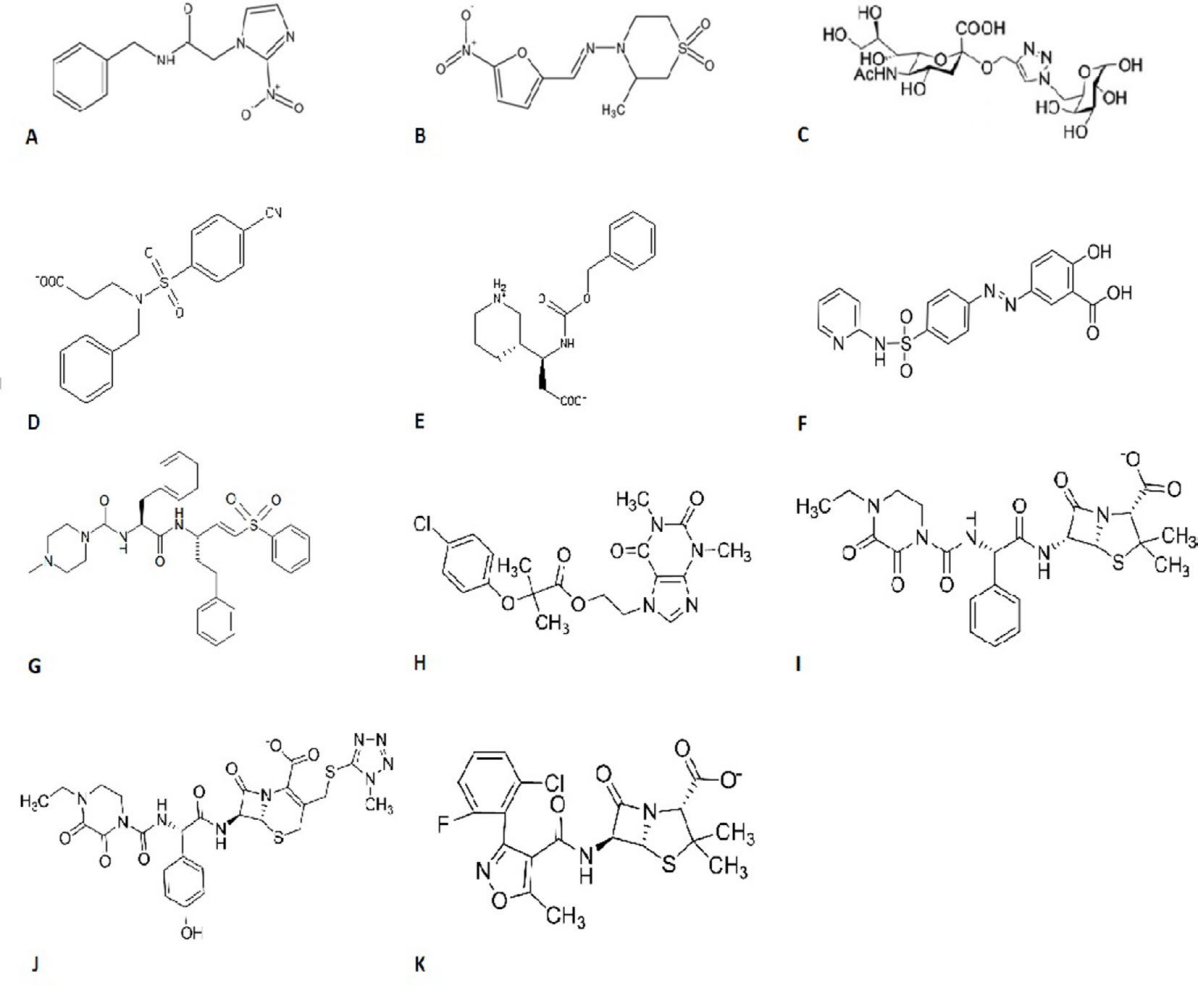
Chemical structures of **(A)** benzonidazole, **(B)** nifurtimox, **(C)** 1,2,3-triazole-linked sialic acid-6-O-galactose and sialic acid-galactopyranoside, **(D)** ZINC13359679, **(E)** ZINC02576132, **(F)** sulfasalazine, **(G)** K777, **(H)** etofylline clofibrate, **(I)** piperacillin, **(J)** cefoperazone, and **(K)** flucloxacillin.

According to the Centers for Disease Control and Prevention (CDC, 2016), the adverse effects caused by the treatment’s great toxicity are mainly gastrointestinal, including vomiting, abdominal pain, anorexia, and weight loss regarding nifurtimox and allergic dermatitis, insomnia, and peripheral neuropathy as related to benzonidazole. Since the discovery of these two compounds, no other drug has been approved for Chagas disease treatment in the last 40 years (Bellera et al., 2015). The treatment is indicated for all individuals with acute infection, cases of congenital infection, immunosuppressed patients, and children with the chronic form of the disease (CDC, 2016).

According to the Drugs for Neglected Diseases Initiative (DNDi, 2015), the current treatment presents issues, such as long-term treatment (30–60 days), dose-dependent toxicity, low adherence rate, and lack of a pediatric formulation. In 2011, a formulation to be used in children under 2 years old produced by the Pharmaceutical Laboratory of Pernambuco (Laboratório Farmacêutico de Pernambuco or LAFEPE) and registered by the National Sanitary Surveillance Agency of Brazil (Agência Nacional de Vigilância Sanitária or ANVISA) was launched with the support of DND*i*. Some of the advantages linked to this formulation are as follows: there is more safety for this age group; it is a tablet readily dispersible in liquids, which facilitates administration; the need for fractionation is only required in special cases (premature infants weighing less than 2.5 kg); and the drug can be safely administered at home throughout the treatment (twice a day for 60 days).

The poor access to medication is another issue related to Chagas disease treatment. Some countries in non-endemic area, such as Spain, have reported the shortage of the drug (Navarro et al., 2012). The shortage was mainly due to the increased demand of the drug that was not being followed by its production (Chaar, 2014). This demonstrates the need of a management plan for Chagas disease treatment, which still remains in a state of neglect.

Besides that, clinical studies with new candidates for Chagas disease treatment requires great time and effort, and diagnosis limitations such as the lack of tools that can demonstrate parasitological cure in the chronic phase have not yet been overcome (Urbina, 2015). The lack of consensus regarding the efficacy of the chronic disease treatment has also been reported both in experimental models and in studies with chronic patients (Marin-Neto et al., 2009). In 2015, a multicenter randomized study with chronic chagasic patients was conducted by researchers in Brazil, Colombia, El Salvador, Argentina, and Bolivia. The study, Benzonidazole Evaluation for Interrupting Trypanosomiasis, has evaluated the efficacy of benzonidazole in preventing the progression to chronic chagasic cardiopathy and death of patients by following 2.854 individuals for 5 years. Although progress has been made regarding the understanding of effect of benzonidazole during treatment, no reduction of heart disease was observed in treated patients (Morillo et al., 2015).

Another clinical trial with Chagas disease patients is the Benznidazole New Doses Improved Treatment & Therapeutic Associations study (DNDi, 2019). The aim of this trial was to find new dosages of the current treatment without decreasing its effectiveness, whereas a combined treatment with fosravuconazole, a broad-spectrum antifungal drug, was also tested. They found that the 2-week treatment is promising as it is four times shorter than the standard treatment, and all patients completed the course. This lower dosage and time of treatment would facilitate treatment adherence, as it would consequently reduce adverse effects. The data of the combination arm (benznidazole combined with fosravuconazole) is still being analyzed.

## Challenges in the Study of Effective Drugs for Chagas Disease

One of the main focuses on Chagas disease research has been the development of an effective control strategy (Bonney, 2014). However, there are some obstacles when considering the development of effective compounds, such as the difficulty of standardizing *in vivo* and *in vitro* tests for their screening and the absence of markers of treatment efficacy and cure criteria for the disease (Romanha et al., 2010; Pinazo et al., 2014). To evaluate the cure criteria, polymerase chain reaction and serologic conversion are used to assess parasite load (Viotti et al., 2014; Torrico et al., 2018). However, serologic conversion may take years to be achieved, so new sensitive markers of efficacy are required (Gonçalves and Novaes, 2018). There are a number of questions that can only be answered by improving experimental research on drugs for Chagas disease, including better *in vivo* models of the disease and knowing what would be the cure criteria for these models (Chatelain and Konar, 2015).

Regarding the treatment, DND*i* has indicated some criteria that are considered acceptable and ideal for Chagas disease as follows: to be active against all strains of *T. cruzi*, the clinical efficacy has to be superior to benzonidazole in all phases of the disease, it must not have contraindications or pharmacologic interactions, and it has to be administered orally (DNDi, 2016).

Therefore, difficulties do exist and there is a need to overcome them to discover a suitable treatment for the disease.

## Synthetic Drugs and New Therapeutic Approaches for Chagas Disease

The search for new drugs against Chagas disease has evolved appreciably in recent years (Buckner and Navabi, 2010). In this context, different strategies of drug design and discovery are used and have proven to be effective, such as molecular simplification, privileged structures use, prodrugs, quantitative structure-activity relationship (QSAR), and molecular docking. Molecular simplification is applied when there is a need to reduce the complexity of a target compound. It is a strategy widely used with natural compounds and derivatives (Wang et al., 2019). A study with indole-pirimidine derivatives achieved by molecular simplification found two of these derivatives that had great potential as candidates for the lead optimization of a new treatment for Chagas disease (Braga et al., 2017). The use of privileged structures that can accept ligands to multiple receptors is another strategy used to find new treatment for neglected diseases, including Chagas disease. Some of these promising structures are thiossemicarbazones and thiazolidinones (Leite et al., 2017). The prodrugs, which are compounds that need to be biotransformed to show its pharmacologic activity, are used to improve pharmacologic (solubility, chemical stability), pharmacokinetic (absorption, metabolism), and pharmacodynamic (decrease toxicity, activation to a reactive agent) features (Abet et al., 2017). A study with three oral E1224 (a water-soluble ravuconazole prodrug) regimens and benznidazole in subjects with chronic indeterminate Chagas disease suggested the use of E1224 in combination with benznidazole (Torrico et al., 2018). QSAR and molecular docking are very useful computational tools to predict the activity of compounds (Ferreira et al., 2015). Both strategies were done to predict the activity of a series of benzimidazole derivatives in which QSAR models conducted the design of these derivatives with improved potency (Pauli et al., 2017).

For studies to progress, different approaches such as rational design and discovery and drug repurposing are done. Some strategies of the aforementioned approach were described above. Drug repurposing relies on finding new uses for marketed therapeutics or substantially characterized investigational compounds (Langedijk et al., 2015). Repurposing programs have mainly relied on bioinformatics and structure-based approaches aside from target phenotypic screens using whole-cell assays (Ferreira and Andricopulo, 2016). Some repositioned drugs that reached clinical trial studies for Chagas disease are the antifungals fexinidazole and posaconazole (Molina et al., 2014; DNDi, 2019).

Drug discovery based on target is the main focus of this revision, which is the rational drug discovery that relies in the search of specific molecular targets of the parasite, most of them being enzymes, and design compounds that can modulate them (Gilbert, 2013; Field et al., 2017). Thus, several molecules have been explored in medicinal chemistry programs applying drug planning methods based on receptor and ligand structures (Dias et al., 2009). Some important *T. cruzi* enzymatic targets such as trans-sialidase, nitroreductase type 1 (NTR), and cruzain were investigated and selected in this review as they are some of the most searched targets for Chagas disease (Miller III and Roitberg, 2013a; Balaña-Fouce et al., 2014; Bermudez et al., 2016; San Francisco et al., 2017; Scarim et al., 2018).

Trans-sialidase is an enzyme that is absent in mammalian cells and is involved in parasite evasion from the immune system, more specifically from the complement system, as well as in adhesion and invasion onto host cells, decrease of T and B lymphocytes, and thymic atrophy (Rubin-de-Celis et al., 2006; Rubin-de-Celis and Schenkman, 2012; Nardy et al., 2016). The sialylmimetic neoglycoconjugates were investigated, where 1,2,3-triazole-linked sialic acid-6-O-galactose and sialic acid-galactopyranoside compound (**Figure 1C**) stood out regarding their anti-enzyme activities (Campo et al., 2012). The conjugation of lactitol analogues with polyethylene glycol has also been shown to be effective in inhibiting this enzyme during *in vitro* studies (Giorgi et al., 2012). More recently, a database screening of more than 4 million compounds has found two molecules, ZINC13359679 (**Figure 1D**) and ZINC02576132 (**Figure 1E**), as the most promising candidates that could inhibit this enzyme (Miller III and Roitberg, 2013b). When computational strategies screened more than 3000 drugs approved by the Food and Drug Administration (FDA), and *in vitro* and *in vivo* tests were performed, it was seen that the anti-inflammatory sulfasalazine (**Figure 1F**) can be used as a lead to design new enzyme inhibitor drugs (Lara-Ramirez et al., 2017). Recently, it was found that molecules containing amide, hydroxyl, and carboxylic acid radicals in their aromatic rings can enhance the biological activity of these enzyme inhibitors, which may lead to a better rational drug design (Kashif et al., 2017).

NTR is an enzyme involved in the activation of nitroheterocyclic compounds, such as benzonidazole and nifurtimox (Wilkinson et al., 2008). Recent studies have shown its *in vitro* and *in vivo* potential in studies of absorption, distribution, metabolism, and excretion of this enzyme inhibitors (Papadopoulou et al., 2013; Papadopoulou et al., 2015; Papadopoulou et al., 2016). Corroborating this idea, a study demonstrated that NTR inhibitors were more effective than inhibitors of ergosterol synthesis when tests using amastigotes from representatives of each discrete type unit (DTU) were performed (Moraes et al., 2014). DTU is a set of genetically similar *T. cruzi* populations that can be identified by common genetic, molecular, and immunologic markers (Tibayrenc, 2003). In this way, different DTUs can present diverse response toward the compounds and this strengthens the use of this approach.

Cruzain, also called cruzipain or GP57/51, is the most abundant protein in the T. cruzi cysteine protease family, being the key enzyme for the parasite’s intracellular replication (Alvarez et al., 2012) and in its escape from the host’s immune system, representing a molecular target to design new antiparasitic drugs (Harth et al., 1993; McKerrow et al., 1999; Siles et al., 2006). In vitro tests performed by our research group with thiazide derivatives aryl thiosemicarbazones and aryl-4-thiazolinones showed that they had an effective anti-T. cruzi activity (Donnici et al., 2009) even on a nanomolar scale as confirmed by the high affinity of these molecules for cruzain during docking studies. The most potent and well-known inhibitor of this enzyme is K777 (**Figure 1G**), which is about to enter clinical studies (McKerrow et al., 2009; Duschak, 2016). In the same way as for trans-sialidase, a screening with drugs that were approved by the FDA was conducted for cruzain and found that four compounds could be used as a basis to design new drugs, namely etofylline clofibrate (antilipemic, **Figure 1H**) and piperacillin, cefoperazone, and flucloxacillin (antibiotics, **Figures 1I–K**, respectively) (Palos et al., 2017).

## Conclusion and Perspectives

Different strategies are applied in view to find an alternative treatment for Chagas disease, including drug repositioning and target-based drug design. Opportunities for drug planning studies are promising, especially with the recent advances in molecular and cell biology, medicinal and computational chemistry, and planned organic synthesis. For a thorough and correct election of a drug candidate against Chagas disease, further activity studies within preclinical trials (such as in vivo testing) and clinical trials are needed. However, there is still a large gap regarding the standardization of these tests as well as a lack of biomarkers and cure criteria. With the combination of different techniques and strategies to discover new drugs, we hope that a refinement in search for a new therapy against Chagas disease will be facilitated, leading to the achievement of this important goal.

## Author Contributions

ACC-S and MCAB-C wrote the manuscript. VRAP, ACCL, and MZH contributed to the discussion of the draft and made final corrections.

## Funding

ACC-S is the recipient of a Ph.D. fellowship from CAPES.

## Conflict of Interest Statement

The authors declare that the research was conducted in the absence of any commercial or financial relationships that could be construed as a potential conflict of interest.
